# Cotton CC-NBS-LRR Gene *GbCNL130* Confers Resistance to Verticillium Wilt Across Different Species

**DOI:** 10.3389/fpls.2021.695691

**Published:** 2021-09-08

**Authors:** Tinggang Li, Qianqian Zhang, Xilong Jiang, Ran Li, Nikhilesh Dhar

**Affiliations:** ^1^Shandong Academy of Grape, Shandong Academy of Agricultural Sciences, Jinan, China; ^2^Institute of Plant Protection, State Key Laboratory for Biology of Plant Diseases and Insect Pests, Chinese Academy of Agricultural Sciences, Beijing, China; ^3^Department of Plant Pathology, University of California, Davis, Salinas, CA, United States

**Keywords:** cotton, fungal disease, verticillium wilt, resistance gene, SA signaling pathway, reactive oxygen species, inducible defense response

## Abstract

Verticillium wilt (VW) is a destructive disease in cotton caused by *Verticillium dahliae* and has a significant impact on yield and quality. In the absence of safe and effective chemical control, VW is difficult to manage. Thus, at present, developing resistant varieties is the most economical and effective method of controlling Verticillium wilt of cotton. The CC-NBS-LRR (CNL) gene family is an important class of plant genes involved in disease resistance. This study identified 141 *GbCNLs* in *Gossypium barbadense* genome, with 37.5% (53 genes) *GbCNLs* enriched in 12 gene clusters (GC01–GC12) based on gene distribution in the chromosomes. Especially, seven *GbCNLs* from two largest clusters (GC11 and GC12) were significantly upregulated in the resistant cultivar (Hai No. 7124) and the susceptible (Giza No. 57). Virus-induced gene silencing of *GbCNL130* in *G. barbadense*, one typical gene in the gene cluster 12 (GC12), significantly altered the response to VW, compromising plant resistance to *V. dahliae*. In contrast, *GbCNL130* overexpression significantly increased the resistance to VW in the wild-type *Arabidopsis thaliana*. Based on our research findings presented here, we conclude that *GbCNL130* promotes resistance to VW by activating the salicylic acid (SA)-dependent defense response pathway resulting in strong accumulation of reactive oxygen species and upregulation of pathogenesis-related (PR) genes. In conclusion, our study resulted in the discovery of a new *CNL* resistance gene in cotton, *GbCNL130*, that confers resistance to VW across different hosts.

## Introduction

Plants are often exposed to various pathogens in the course of their existence. Since plants are immobile, they rely on their immune system to resist infection from numerous pathogens (Zhou et al., 2017). Besides the natural barriers to prevent pathogen infection, such as epidermal villi, waxy layers, and cuticles, plants also rely on two highly efficient inducible defense systems (Jones and Dangl, 2006). PAMP-triggered immunity (PTI) is the first layer of the plant defense system, and it is triggered upon recognition of pathogen-associated molecular patterns by pattern recognition receptors (PRRs; Dangl et al., 2013). When adaptive pathogens suppress or bypass the PTI immune response through the secretion of virulent effectors, plants activate the second layer of the defense, effector-triggered immunity (ETI), to counter the pathogen infection (Césari et al., 2014). ETI is a specific recognition response to unique pathogen effectors (Ameline-Torregrosa et al., 2008). ETI-mediated immune response is overcome by the pathogens due to rapid evolution of new effectors to counter the increased host resistance (Yu et al., 2014). Therefore, identifying plant resistance genes provides a basis to not only investigate disease resistance mechanisms but also deploy candidate genes to develop disease-resistant crop varieties.

Several resistance genes have been identified and divided into at least five classes based on different protein domains (Yang and Wang, 2016). However, the Nucleotide Binding Site-Leucine-Rich Repeat (NBS-LRR) group of genes, consisting of an N-terminal variable region, a central NB-ARC/NBS, and a C-terminal Leucine-Rich Repeat (LRR) domain remains the dominant class of genes involved in disease resistance (Takken and Goverse, 2012). The NBS-LRR proteins can further be divided into two sub-classes based on the *N*-terminal structural diversity, TIR-NBS-LRR (TNL) containing the toll and interleukin-1 receptor (TIR) domain and CC-NBS-LRR (CNL) with coil-coil (CC) domain at the N-terminal (Meyers et al., 1999). Gene duplication and amplification cause extensive rearrangement and distribution of *NBS-LRR* genes in the genome (Hulbert et al., 2001; Meyers et al., 2003; Li et al., 2010). A gene cluster is a genetic variation library of the resistance (*R*) gene through gene replication, recombination, gene transformation, and diversity selection, and can be conducive to the evolution of the structural diversity and specificity of the resistance genes (Meyers et al., 2003; Ameline-Torregrosa et al., 2008; Guo et al., 2011).

The *NBS-LRR* genes are involved in recognizing various plant pathogen effectors through gene interactions (Yang and Wang, 2016). The C-terminal variable LRR domain of the resistance proteins can specifically recognize a cognate pathogen effector bypassing the PTI immune response (Martin et al., 2003). The NBS domain is a molecular switch essential in the binding and hydrolysis of ATP and GTP during antagonistic interactions between plants and pathogens *via* several specific motifs, including Walker A, Walker B, Apaf-1, R proteins and CED-4 (ARC1), ARC2, and Methionine-Histidine-Aspartate (Tameling et al., 2002; Williams et al., 2011). The N-terminal end of resistance proteins contains either the TIR or CC domain, determining various signal components, such as *Enhanced Disease Susceptibility 1* (*EDS1*) or *Non-race-specific Disease Resistance 1* (*NDR1*), essential in downstream signaling activation (Feys and Parker, 2000; Ade et al., 2007). Eventually, the host develops several resistance responses, including salicylic acid (SA) biosynthesis leading to upregulation of pathogenesis-related (PR) genes, and generation and sustained amplification leading to rapid accumulation of reactive oxygen species (ROS), and other antimicrobial compounds to prevent further spread of the pathogen (Heath, 2000).

Verticillium wilt (VW) in cotton is caused by *Verticillium dahliae*. VW can affect at least 60% of cotton planting area, significantly reducing yield and quality (Li et al., 2018). Currently, breeding of resistant varieties is the best method of preventing and controlling VW (Li et al., 2017b). New resistance genes should be continuously identified as pathogens evolve *via* the gene-for-gene hypothesis (Flor, 1971). Besides, the mechanism of the resistance gene’s function should be elucidated to devise and deploy effective control measures, including the development of cotton disease-resistant varieties and implement effective cultural practices to limit the losses from pathogen infection (Yang and Wang, 2016; Li et al., 2018). Currently, only a few resistance genes to VW, including *Gossypium arboreum ribosomal protein L18* (*GaRPL18*), *G. barbadense NB-ARC domain-containing 1* (*GbaNA1*), *G. barbadense* nucleoredoxin 1 (*GbNRX1*), *G. barbadense Receptor-Like Kinase* (*GbRLK*), *G. barbadense subtilase 1* (*GbSBT1*), *G. barbadense Ser/Thr protein kinase* (*GbSTK*), *G. barbadense thaumatin-like protein 1* (*GbTLP1*), *G. barbadense Ve1 like protein* (*Gbve1*), *Gossypium hirsutum polyamine oxidase* (*GhPAO*), *G. hirsutum villin 4* (*GhVLN4*), and *G. hirsutum polygalacturonase-inhibiting protein 1* (*GhPGIP1*), have been explored (Munis et al., 2010; Zhang et al., 2012, 2013; Zhao et al., 2013; Mo et al., 2015; Duan et al., 2016; Li et al., 2016, 2017a; Gong et al., 2017; Liu et al., 2017; Dhar et al., 2021; Ge et al., 2021). Besides *G. barbadense* resistance to *V. dahliae* (*GbRVd*), some reports have indicated that CNL proteins are essential in cotton resistance to VW (Yang et al., 2016). Resistance genes in cotton activate a variety of defense responses after *V. dahliae* infection, including regulating the levels of defense hormones [salicylic acid (SA), jasmonic acid (JA), etc.], which potentiate the oxidative burst and activate the expression of pathogenic genes, so as to fortify the resistance of cotton plants to *V. dahliae* (Zhang et al., 2013; Duan et al., 2016; Gong et al., 2017; Liu et al., 2017; Dhar et al., 2021). For instance, in *G. hirsutum*, the silencing of *G. hirsutum wall-associated kinase-like* (*GhWAKL*) gene resulted in the decrease of salicylic acid content, inhibited the defense response mediated by salicylic acid, and thus increased the susceptibility of cotton to *V. dahliae* (Feng et al., 2021); *G. hirsutum dominant suppressor of camta3 1* (*GhDSC1*) mediates *V. dahliae* resistance through jasmonate signaling pathway, which is also related to the accumulation of ROS and the increased expression of pathogenesis-related genes (Li et al., 2019).

While there are more than 50 species of cotton occurring across the globe, *G. hirsutum* alone accounts for more than 90% of the cultivated cotton worldwide. However, resistance (*R*) genes to VW are yet to be discovered in this cultivated species (Li et al., 2014). The completion of high-quality genome sequencing and annotation has provided a basis for identifying VW resistance genes in *G. barbadense* (Wang et al., 2019). The objectives of the current study were designed to: (1) identify the *GbCNL* genes in *G. barbadense* genome, (2) screen and identify the candidate *CNL* gene(s) involved in VW resistance, (3) functional characterization of the candidate gene in *Arabidopsis thaliana* to confirm its role in VW resistance, (4) determine the hormone signaling pathway involved in VW disease resistance mediated by the putative candidate gene, and (5) investigate various aspects of defense responses conferred by the candidate *R* gene.

## Materials and Methods

### Identification of CNL Family in *G. Barbadense*

Genome data of *G. barbadense* were downloaded from^1^ (Wang et al., 2019). HMMER program was used to match NB-ARC proteins based on the Hidden Markov Model profile of NB-ARC domain (PF00931) in *G. barbadense* genome with an E-value <1e^−5^ with Z-score >25 (Finn et al., 2016). PfamScan^2^ and Paircoil2^3^ were used to verify the presence of LRR domains and CC motifs (Lupas et al., 1991; McDonnell et al., 2006), respectively. SMART and InterProScan databases were used to further verify the conserved domains (Hunter et al., 2012; Letunic and Bork, 2018). Candidate genes encoded with NB-ARC, LRR, and CC domains/motifs were defined as *GbCNLs* in *G. barbadense*. Finally, the *GbCNLs* were anchored to *G. barbadense* genome by MapInspect software (Li et al., 2018). Gene clusters definition standards were implemented in accordance with the standards formulated previously (Christie et al., 2016).

### Expression Pattern of CNL Family Genes

The expression level of CNL family genes in *G. barbadense* response to *V. dahliae* (5×10^6^ conidia/ml) was collected from our previous published transcriptome data to evaluate the expression pattern of CNL family genes, roots treated with sterile distilled water were set as control, and the raw RNA-seq data pertaining to the same were deposited into NCBI public database at the sequence read archive under accession NO. SRP03537 (Chen et al., 2015). The candidate CNL family genes were further detected on the *G. barbadense* response to *V. dahliae*. Briefly, *V. dahliae* isolate *Vd991* (highly virulent strain) was cultured in complete medium (CM) at 25°C condition for 5days (Chen et al., 2016). The conidia were then collected in deionized water to adjust its concentration to 5×10^6^ conidia/ml, and three-week-old cotton plants (resistance cultivar Hai No. 7124 and susceptible cultivar Giza No.57) were inoculated with *V. dahliae* using the root-dip method. The cotton roots were harvested at seven time points (0, 6, 12, 24, 48, 72, and 120h), flash frozen for RNA extraction later. The expression level of candidate CNL family genes was detected using RT-qPCR, and cotton *18S* gene (L24145.1) was set as internal control. 2^−ΔΔCT^ method was used to evaluate the expression levels (Livak and Schmittgen, 2001). Primers used for the expression analysis are listed in Supplementary Table S1.

### Virus-Induced Gene Silencing in *G. Barbadense*

The specific fragments of candidate genes were amplified from *G. barbadense* cDNA and integrated into the vector *pTRV2*, which was then transferred into *A. tumefaciens* GV3101. *A. tumefaciens* containing *pTRV1* and *pTRV2* plasmid were mixed in a ratio of 1:1 and co-infiltrated into the two-week-old *G. barbadense* Hai No. 7124 plants. The validity of virus-induced gene silencing (VIGS) system was tested by *Chloroplastos alterados 1* (*CLA1*) gene, and the silencing efficiency of candidate genes was tested when white leaf phenomenon was observed in *CLA1* gene-silenced plants. The disease resistance was also identified by root-dip method, and the disease symptoms were observed after 20days. Degree of disease progression was evaluated by the percentage of leaves wilting after infection, and the fungal colonization was quantified by *V. dahliae Elongation Factor 1-a* (*EF-1a*, XM_009652412.1) and the cotton *18S* gene. Six plants for each assay were used, and the experiment was repeated three times with similar results.

### Transgenic *A. Thaliana* and Resistance Assay

Based on the genome of *G. barbadense*, primers were designed according to Gbar_D11G034490 sequence to amplify the full-length fragment of *GbCNL130* gene (Supplementary Table S2). *GbCNL130* gene fragment was integrated into binary vector *pCAMBIA1304* and introduced into *A. tumefaciens* GV3101. *A. thaliana* wild-type Col-0 was transformed by *Agrobacterium*-mediated floral dip method as described previously (Clough and Bent, 1998). PCR and RT-PCR were performed using gDNA and cDNA to identify T_3_ homozygous transgenic plants using wild-type Col-0 as the control; *A. thaliana ubiquitin 5* (*UBQ5*, NM_116090.3) was used as the internal control gene for RT-PCR analysis. Primers are listed in Supplementary Table S1. For resistance assay, 3-week-old plants were inoculated with the *V. dahliae* conidia by root-dip method by dipping in 5×10^6^ conidia/ml *V. dahliae* for 5min and then transplanted into vermiculite soil. The phenotype of transgenic plants resistant to *V. dahliae* was recorded at two-week post-inoculation. The development of fungal biomass in plant tissue was determined by qPCR that performed using SYBR premix Ex Taq II kit (TaKaRa, Japan) with primers specific to the *A. thaliana UBQ5* gene and *V. dahliae Elongation Factor* 1-a (*EF-1a*).

### ROS Accumulation Detection

Two-week-old *A. thaliana* leaves were infiltrated with 10μl conidia suspension of *V. dahliae*, sterile water as a control, and 3,3'-diaminobenzidine (DAB) solution was used to detect the accumulation of ROS at 12h post-inoculation. The leaves were vacuum infiltrated with the DAB buffer at 25°C in the dark. The reaction was terminated after 12h, and the leaves were then washed clean with sterile water. The leaves were washed with 75% ethanol (v/v) to remove the chlorophyll pigments followed by three washes with deionized water to remove the excess ethanol. Finally, the leaves were suspended in 30% glycerol (v/v). The resulting brown coloration of the leaves was quantified using the ImageJ software to determine the accumulation of ROS.

### Gene Expression Analysis

*Arabidopsis thaliana* wild-type *Col-0* and *GbCNL130* transgenic line was inoculated with *V. dahliae* conidia suspension using the root-dip method. Root samples were collected from each treatment at 24h after inoculation, in triplicates. The PLANTeasy kit (YPH-Bio, Beijing, China) was used for extracting total RNA, and cDNA synthesis was performed using the TIANSeq M-MLV (RNase H-) reverse transcriptase (Tiangen, Beijing, China). RT-qPCR analysis was conducted using the SYBR Premix Ex Taq kit (TaKaRa, Kusatsu, Japan) with a QuantStudio 6 Flex qPCR System (Applied Biosystems, Foster City, CA, United States). The relative expression level of salicylic acid (SA) and jasmonic acid (JA) signaling genes was detected in the transgenic lines overexpressing *GbCNL130* and the control wild-type Col-0 plants. Five plants for each treatment in three replicates were used for the gene expression analysis. The relative expression levels were determined using the 2^−ΔΔCT^ method as described earlier (Livak and Schmittgen, 2001). Primers used for the above study are listed in Supplementary Table S1.

## Results

### Identification of *GbCNLs* in *G. Barbadense* Genome

**Figure 1:**
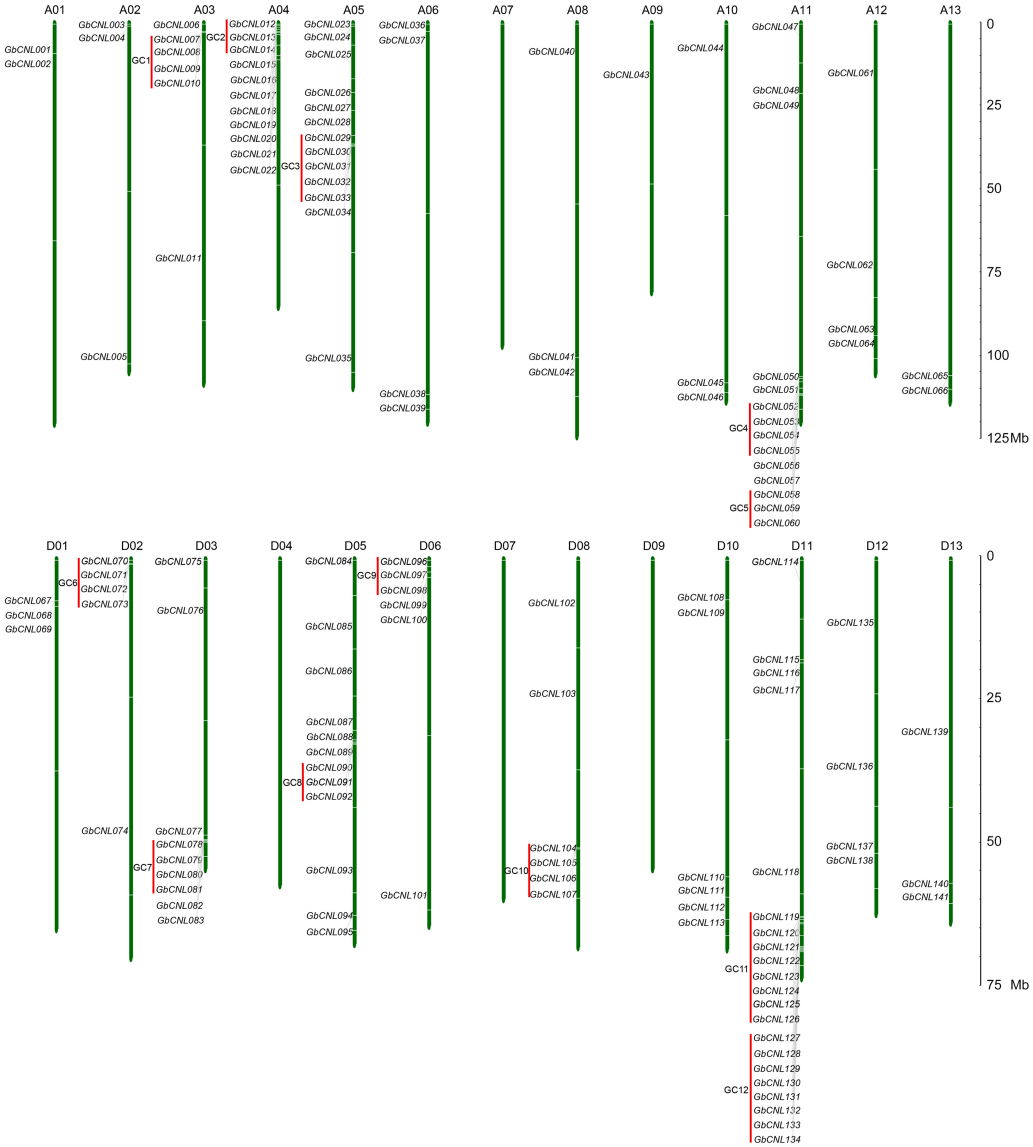
Chromosome locations of the *GbCNL*s on *Gossypium barbadense* genome. Green lines represent the chromosome and chromosome numbers are indicated at the top of each vertical bar; red lines represent the enrichment clusters of *GbCNL*s. The scale on right represents the chromosome length in megabase (Mb).

With the identification of conserved domains/motifs in the encoding proteins from *G. barbadense* genome, a total of 141 proteins were defined as CNL family (*GbCNLs*), each containing the CC, NB-ARC, and LRR domains (Supplementary Table S3). Predicted analysis showed that *GbCNLs* were distributed on 22 chromosomes of *G. barbadense* genome, except for the chromosomes of A06, D04, D07, and D09 (Figure 1). Here, *GbCNL*s were named according to their chromosomes order from A01 to D13 (*GbCNL001*–*GbCNL141*). The distribution of *GbCNLs* is in disequilibrium, 11 chromosomes only recruited less than or equal to four members and randomly located these chromosomes, but other 10 chromosomes (except for D10) enriched high abundance of *GbCNL*s. For instance, chromosome A04, A05, A11, D05, and D11 had 11 (7.8%), 13 (9.2%), 14 (9.9%), 12 (8.5%), and 21(14.9%) genes (Figure 1), respectively. Further, identification of the *GbCNL*s locations on chromosomes showed that many *GbCNL*s occurred in gene clusters, in a total of 12 gene clusters (GC1–GC12) accounting for 37.5% (53 genes) of the predicted *GbCNLs* (red lines in Figure 1). Especially, two gene clusters, GC11 and GC12, had linkage characteristics on D11 chromosomes, and both contained eight *GbCNLs* (*GbCNL119*–*GbCNL126* and *GbCNL127*–*GbCNL134*). Taken together, these results suggest that the *G. barbadense* genome may encode for a large number of CNL family proteins, with characteristics of gene clusters enrichments.

### *GbCNLs* in GC11 and GC12 Cluster Are Involved in Resistance Against *Verticillium* Wilt in Cotton

**Figure 2:**
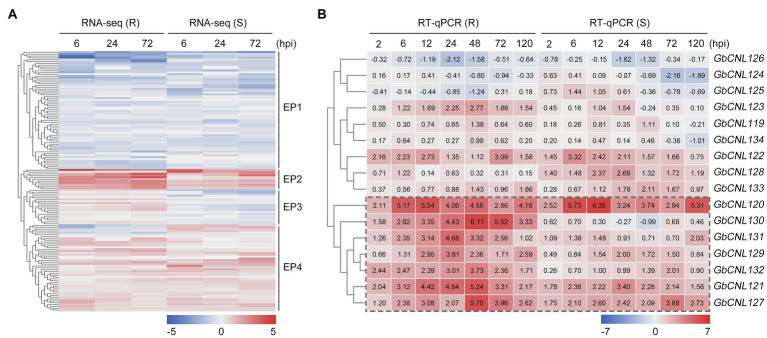
The expression pattern of *GbCNL*s in resistant (cv. Hai No. 7124) and susceptible (cv. Giza No. 57) *Gossypium barbadense* cultivar inoculated with *V. dahliae*. **(A)** RNA-seq showed the expression level of *GbCNLs* at 6, 24, and 72h time points between the control and *V. dahliae*-treated samples. Box in dotted line represents the *GbCNLs* with upregulation pattern common to both resistant and susceptible cotton cultivars. Hpi, hours post-inoculation. **(B)** RT-qPCR analysis of expression level of *GbCNL*s from GC11 and GC12 at 2, 6, 12, 24, 48, 72, and 120h points between the control *versus V. dahliae*-treated samples. Number in box represents the value of fold change that was calculated from three independent replicates. Box in red and blue color represents the upregulation and downregulation, respectively. Box in dotted line represents the *GbCNL*s from GC11 and GC12 cluster that is significantly upregulated in the resistant cotton cultivar in response to *V. dahliae*.

To investigate the role of CNL in Verticillium wilt resistance in cotton, we analyzed the expression patterns of the predicted *GbCNLs* from the root transcriptome data in response to *V. dahliae* treatment (including the resistant *G. barbadense* cv. Hai No. 7124, and the susceptible *G. barbadense* cv. Giza No. 57) reported in our earlier study (Chen et al., 2015). Analysis of transcript data showed that the gene expression pattern of *GbCNLs* displays significant different between the resistance and susceptible cultivar, and for few genes, the response pattern to *V. dahliae* is similar (Supplementary Table S4). Further analysis of transcription level revealed that the expression pattern (EP) of *GbCNLs* was clustered in four groups (EP1–EP4; Figure 2A). EP1 cluster contains 64 *GbCNLs* that showed downregulation at three different time points (6, 24, and 72h) in both the resistance and susceptible cotton cultivars, and while the EP2 cluster with 11 *GbCNLs* displayed upregulation. Furthermore, EP3 cluster contains 19 *GbCNLs* that are upregulated in the resistant cotton cultivar (Hai No. 7124), but were downregulated in the susceptible cotton cultivar (Giza No. 57); however, the time points of differential expression change were not significant (less than two-fold change). Finally, the EP4 cluster of 47 *GbCNLs* shows the maximum contrast in expression between resistance and susceptible cotton (Figure 2A). Notably, the expression change in most *GbCNLs* in EP2 cluster was more significant in the resistant cotton cultivar (Hai No. 7124) in comparison with the susceptible cotton cultivar (Giza No. 57), with six genes in this EP2 cluster (*GbCNL121*, *GbCNL122*, *GbCNL127*, *GbCNL130*, *GbCNL131*, and *GbCNL132*) belong to two largest gene clusters of GC11 and GC12 (Figure 2A), which indicated that GC11 and GC12 may be involved in the resistance response of cotton against *V. dahliae*. Therefore, we further analyzed the expression level of *GbCNLs* from GC11 and GC12 clusters in the resistant and susceptible cotton cultivar during the progression of *V. dahliae* infection (seven time points; Supplementary Table S5). RT-qPCR analysis showed that seven out of 16 *GbCNLs* in GC11 and GC12, including *GbCNL120, GbCNL121, GbCNL127, GbCNL129, GbCNL130, GbCNL131*, and *GbCNL132*, were expressed significantly at higher levels in the resistant cultivar Hai No. 7124 in comparison with the susceptible cultivar Giza No. 57 (Figure 2B), which is in line with the analysis of the transcriptome results (Figure 2A). Especially, *GbCNL130* displayed significantly higher fold change in the resistant Hai No. 7124 cultivar (2.11–5.54-fold) than in the susceptible cv. Giza No. 57 (−0.99–0.68-fold) upon infection with *V. dahliae* (Figure 2B). Together, these results suggested that in cotton, the *GhCNLs* in two largest cluster (GC11 and GC12) on D11 chromosome may be involved in the resistance response against *V. dahliae*.

### *GbCNLs130* Is a Candidate Gene Confers Verticillium Wilt Resistance in Cotton

**Figure 3:**
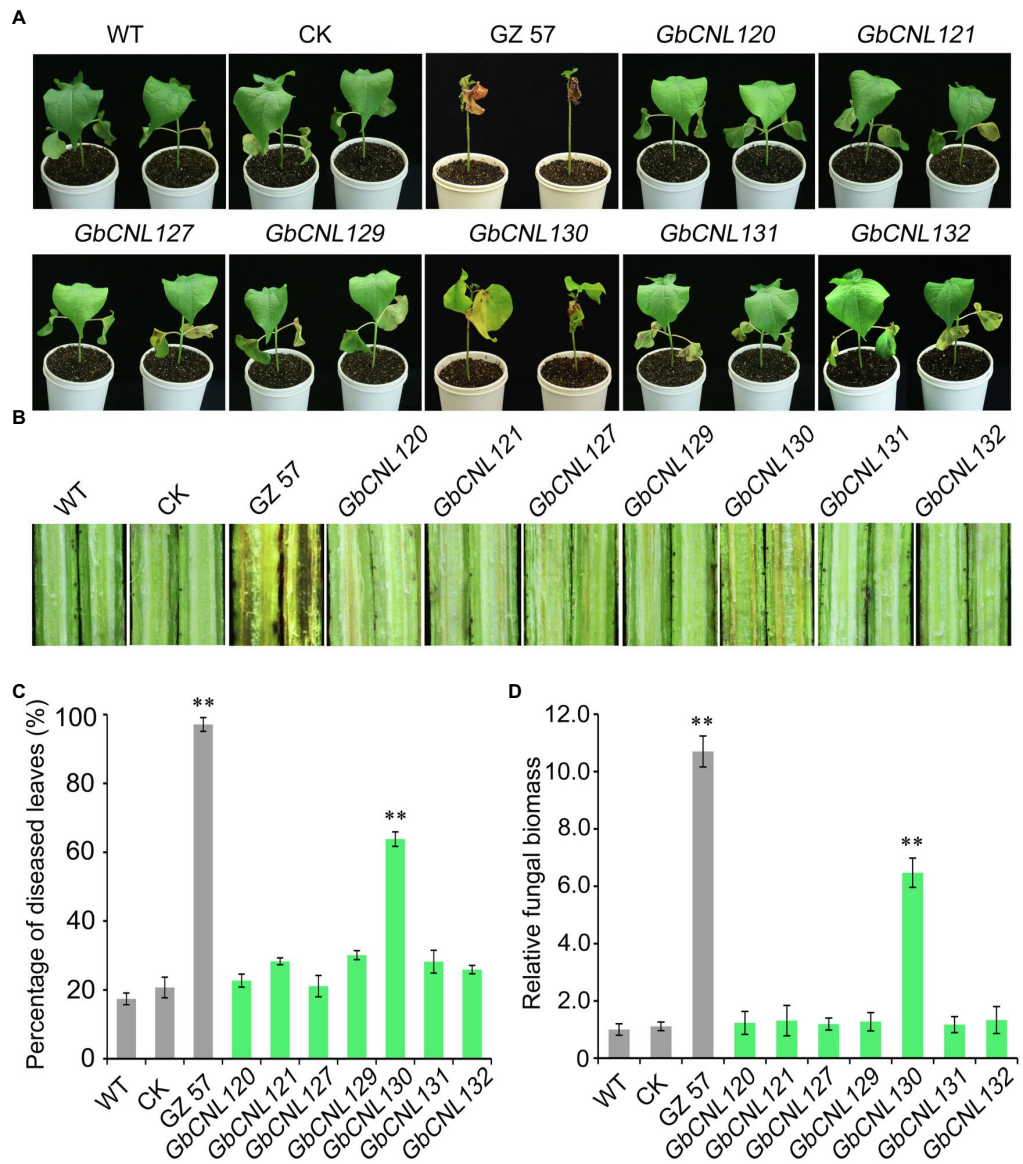
Virus-induced gene silencing (VIGS) analysis of seven candidate *GbCNLs* genes associated with *V. dahliae* resistance. After 21days post-gene silencing upon VIGS treatment, the control *pTRV2:00* (CK), wild-type (WT), and gene-silenced plants were evaluated for VW (disease) resistance using the root-dip method. **(A)** Phenotype of wilting leaves on the *GbCNLs*-silenced cotton plants, treated plants were photographed 4weeks after inoculation with *V. dahliae* (5×10^6^ conidia/mL). **(B)** Phenotype of vascular discoloration. **(C)** Relative ratio of leaves wilting in various genotypes tested. The disease incidence for the susceptible cv. Giza No. 57 was set at maximum. **(D)** Detection of the fungal biomass develop on the *GbCNLs*-silenced cotton plants by RT-qPCR. cv. Giza No. 57 (GZ 57) was used as a negative control. The double asterisk (**) indicates a significant difference at *p*<0.01.

Further, we employed the tobacco rattle virus-based VIGS technology to analyze the Verticillium wilt resistance candidate genes, including the seven *GhCNLs* (*GbCNL120, GbCNL121, GbCNL127, GbCNL129, GbCNL130, GbCNL131*, and *GbCNL132*) in GC11 and GC12 clusters that are significantly upregulated in resistant cotton cultivar in response to *V. dahliae* infection (Figure 2B). The cotton gene *CLA1* is essential for chloroplast development, which confirmed the efficacy of the VIGS approach in the cotton cv. Hai No. 7124 as a result of the *CLA1* silencing in cotton plants resulting in an albino phenotype of the newly emergent leaves (Supplementary Figure S1A). Further, the *GbCNLs*-silenced cotton plants were generated, and analysis of gene expression level showed that all seven *GbCNLs* efficiently silenced 2weeks after VIGS treatment (Supplementary Figure S1B). Thus, we subjected all the *GbCNLs*-silenced cotton plants to *V. dahliae* infection by root-dip method to determine the progression of VW and subsequent resistance response to the causal pathogen. Our results show that only *GbCNL130*-silenced cotton plants were compromised in resistance to *V. dahliae* (Figure 3A; Supplementary Figure S2), with vascular discoloration to an extent similar to the susceptible cultivar of cv. Giza No. 57 (Figure 3B). Further, the extent of susceptibility in the *GbCNL130*-silenced cotton plants was accompanied by high percentage of wilting leaves and increased fungal biomass accumulation similar to the susceptible cultivar of cv. Giza No. 57 (Figures 3C,D); however, individual silencing of five other *GbCNLs* resulted in transgenic cotton plants with no significant alteration in resistance to *V. dahliae* when compared to the wild-type and control plants (Figure 3). Therefore, we conclude that *GbCNL130* is a candidate gene for Verticillium wilt resistance in cotton.

### Ectopic Overexpression of *GbCNL130* in *Arabidopsis* Leads to Increased Resistance to Verticillium Wilt

**Figure 4:**
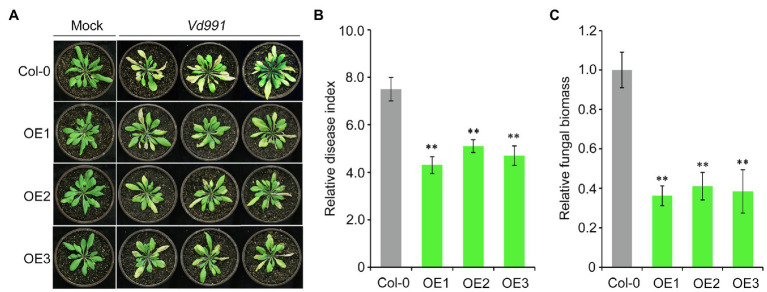
*GbCNL130* enhances *A. thaliana* resistance to *V. dahliae*. **(A)** Phenotype of three independent 35S::*GbCNL130* transgenic lines (OE1, OE2, and OE3) in comparison with the *A. thaliana* wild-type Col-0 after inoculation with *V. dahliae* (5×10^6^ conidia/mL). **(B)** Relative disease index of *A. thaliana* Col-0 and OE lines. **(C)** RT-qPCR detection of relative fungal biomass accumulation in 35S::*GbCNL130* overexpression lines and wide-type Col-0. Double asterisk (**) indicates a significant difference at *p*<0.01.

Next, we further investigated the role of *GbCNL130* in conferring resistance to Verticillium wilt by ectopic overexpression of this candidate gene in the model plant *Arabidopsis*. *Agrobacterium*-mediated transformation was used to introduce the *GbCNL130* into wild-type *A. thaliana*, and several *GbCNL130* overexpression (OE) lines were obtained. Three such independent transgenic lines (OE1, OE2, and OE3) were used to evaluate Verticillium resistance in this study. As expected, all three transgenic lines display heightened resistance to *V. dahliae* infection, with the accompanying symptoms of leaf chlorosis and wilting significantly reduced relative to the wild type of *A. thaliana* Col-0 (Figure 4A). Leaf wilting was significantly alleviated in the transgenic lines overexpressing *GbCNL130* (Figure 4B). Correspondingly, the fungal biomass accumulation of *V. dahliae* in these transgenic lines was also significantly lower than in wild-type plants (Figure 4C). Taken together, these results suggest that *GbCNL130* plays a role in resistance against Verticillium wilt in general, and its role in defense response against *V. dahliae* is conserved across multiple species.

### *GbCNL130*-Mediated Resistance to Verticillium Wilt Involves Salicylic Acid but Not Jasmonate Signaling Pathway

**Figure 5:**
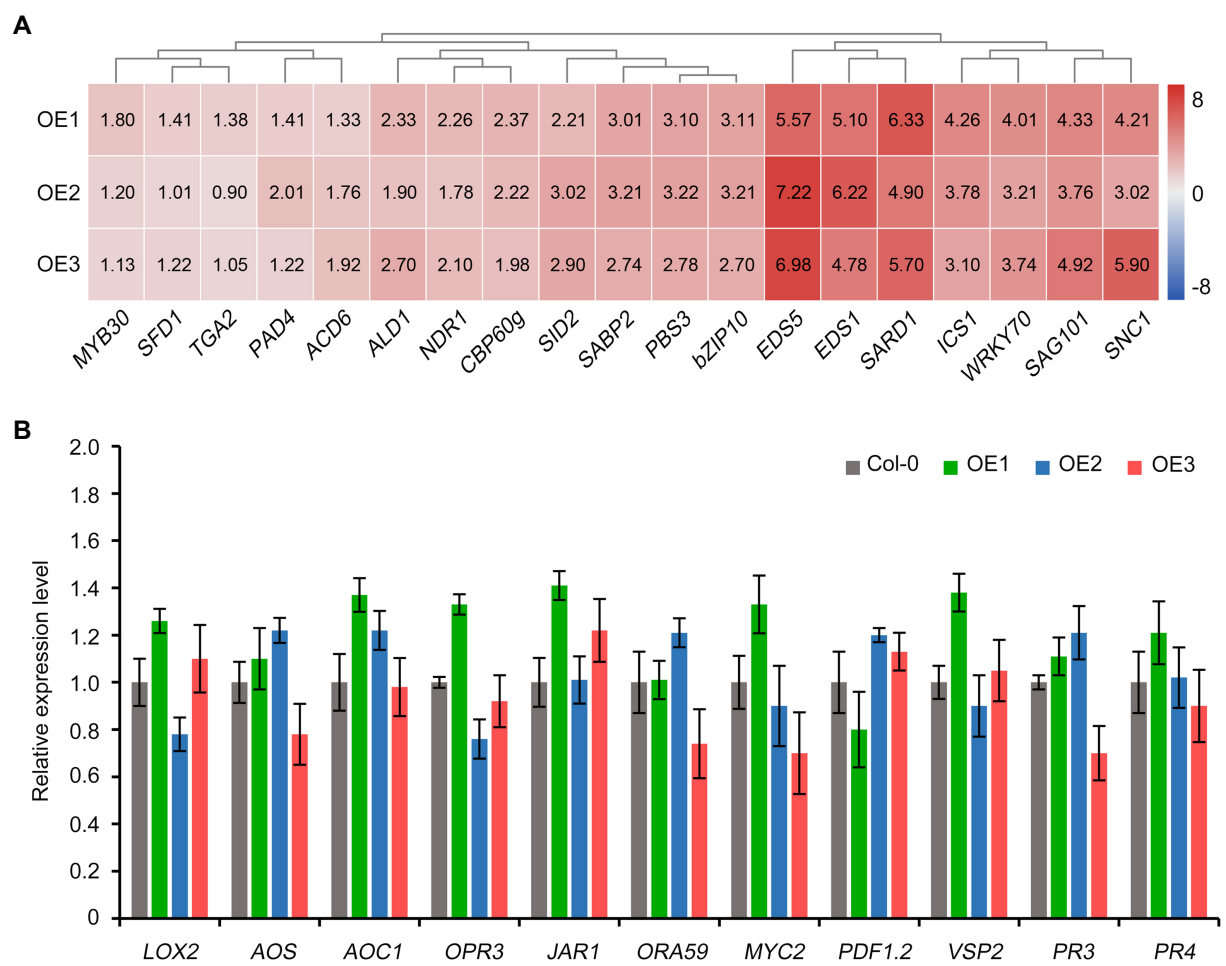
*GbCNL130* regulates SA but not JA signaling pathway genes to confer resistance to *V. dahliae*. Wild-type Col-0 and 35S::*GbCNL130* transgenic lines (OE1, OE2, and OE3) in *Arabidopsis* were inoculated with *V. dahliae* conidia (5×10^6^ conidia/mL) by root-dip method. The root samples were collected 24h after inoculation for RNA extraction and RT-qPCR analysis. **(A)** Heatmap of SA signaling pathway gene expressions in 35S::*GbCNL130 Arabidopsis* plants. Values represent the relative expression of SA signaling pathway genes in three transgenic lines compared to wild-type Col-0 that set as 1.0. **(B)** Detection of JA signaling pathway gene expressions in 35S::*GbCNL130* transgenic lines compared to the wild-type *Arabidopsis* (Col-0).

Salicylic acid (SA) and jasmonic acid (JA) are among the major hormones involved in plant defense response to multiple biotrophic and necrotrophic pathogens (Yoder and Scholes, 2010; Dhar et al., 2020). Therefore, we investigated the expression of various genes involved in SA and JA signaling pathways in *Arabidopsis* upon the ectopic expression of the cotton *GbCNL130*. Surprisingly, the genes involved in SA biosynthesis and signaling were significantly activated in the *Arabidopsis* transgenic lines overexpressing the cotton *GbCNL130* (Figure 5A). However, the expression of the JA biosynthesis and signaling genes did not display any significant alteration in these transgenic lines (Figure 5B). SA signaling plays a critical role in resistance during the early stages of progression of the Verticillium wilt (the biotrophic phase of pathogen growth) in the host plant (Dhar et al., 2020). Thus, the activation of SA signaling but not JA signaling in the transgenic (35S::*GbCNL130) Arabidopsis* plants suggested that *GbCNL130* most likely mediated the resistance to Verticillium wilt at an early infection stage in the host plants.

### *GbCNL130*-Mediated Resistance to Verticillium Wilt Is Due to the Activation of *Pathogenesis-Related* (*PR*) Genes and Accumulation of ROS

**Figure 6:**
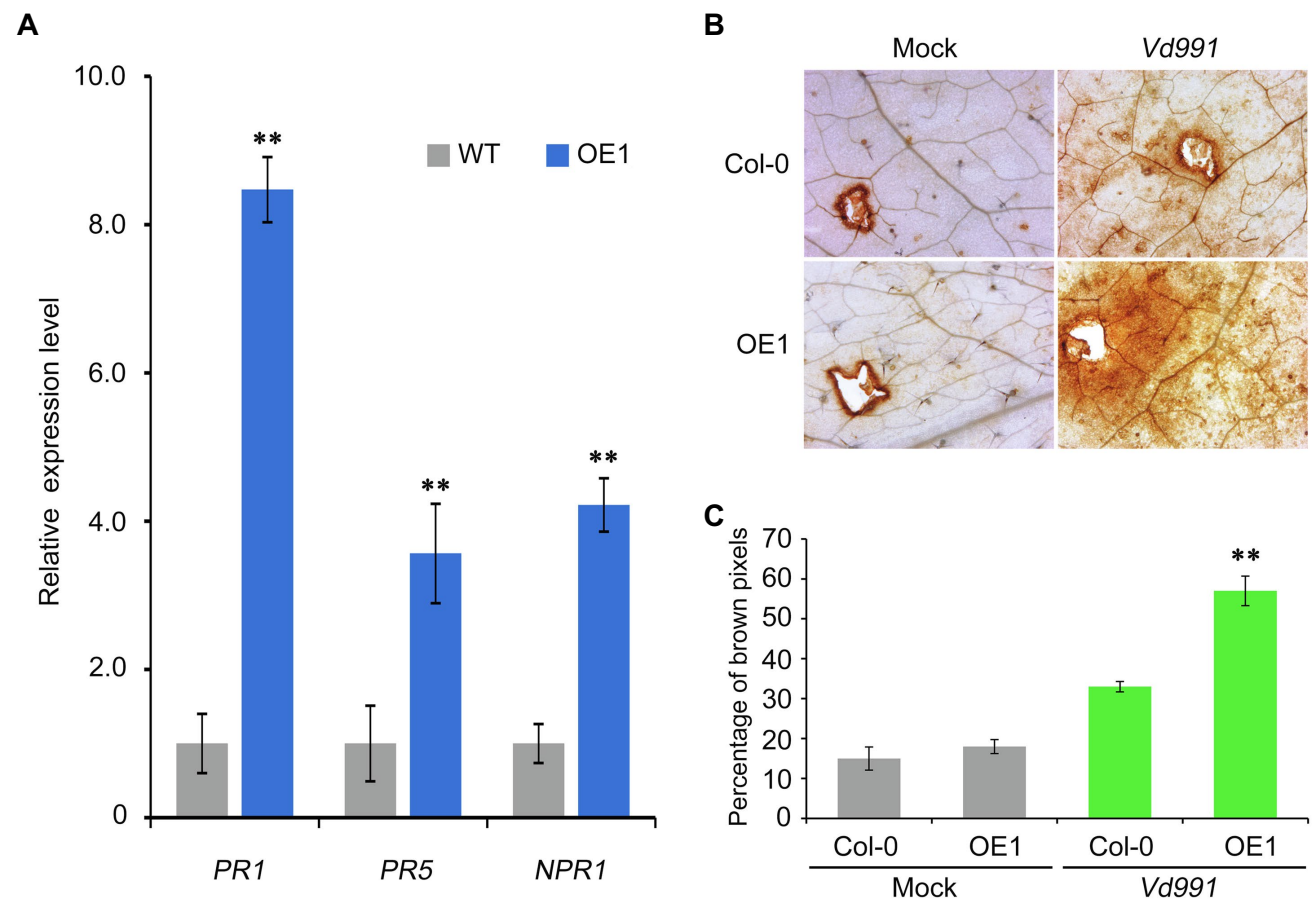
*GbCNL130* mediates the Verticillium wilt resistance by activation of pathogenesis-related genes and accumulation of ROS. **(A)** Expression analysis of SA receptor *NPR1* and marker genes (*PR1* and *PR5*) in the transgenic line OE1 compared to the wild-type Col-0. The root samples for RT-qPCR analysis were collected from the inoculated plants at 48h post-inoculation with *V. dahliae* conidia (5×10^6^ conidia/mL). The double asterisk (**) indicates a significant difference at *p*<0.01. **(B)** Investigation of the ROS accumulation on transgenic line (OE1) compared to the wild-type Col-0. **(C)** Detection of increased ROS accumulation evident from the increased intensity of the brown DAB-stained leaves from the pathogen-treated 35S::*GbCNL130* transgenic lines (OE1) in comparison with the wild-type Col-0. Leaves from 2-week-old plants were inoculated with the *V. dahliae* conidia (5×10^6^ conidia/mL), and the ROS accumulation was detected by DAB staining at 12h post-inoculation. Control leaves were inoculated with deionized water (Mock).

To further confirm that the *GbCNL130*-mediated resistance to Verticillium wilt is due to the upregulation of SA signaling, we checked the levels of the SA receptor gene of *Non-expressor of Pathogenesis-Related gene 1* (*NPR1*), and the SA pathway marker genes, including *Pathogenesis-Related* genes (*PR1* and *PR5*; Wu et al., 2012; Riera et al., 2018). Indeed, both *NPR1* and the SA pathway marker genes *PR1* and *PR5* were significantly upregulated in the 35S::*GbCNL130* plants challenged with *V. dahliae* (Figure 6A), further confirming that the *GbCNL130*-mediated Verticillium wilt resistance is through the activation of the salicylic acid signaling pathway. Since the activation of the SA signaling pathway during biotic stress in plants is accompanied by oxidative bursts originating in different cellular compartments (Wrzaczek et al., 2013), we analyzed the levels of ROS accumulation in the 35S::*GbCNL130* transgenic lines after inoculation with *V. dahliae*. As expected, the 35S::*GbCNL130* transgenic line (OE1) displayed higher and rapid ROS accumulation compared to the wild type of *Arabidopsis* (Col-0) at 12h post-inoculation (Figures 6B,C). Taken together, our results show that *GbCNL130*-mediated resistance to Verticillium wilt involves activation of SA pathway in tandem with the accumulation of ROS.

## Discussion

Cotton is an important economic crop and is severely affected by VW, a typical soil-borne vascular disease (Li et al., 2018). The VW-infected cotton leaves lose turgor, turn yellow, wilts, and eventually die, leading to significant reduction in cotton production or in extreme cases result in total yield loss (Li et al., 2017a). In the light of reduction/ban in the deployment of harmful chemical control methods against this difficult to eradicate soil-borne pathogen, it is more important now than ever before to discover new genetic loci that could fortify resistance to *V. dahliae*. Furthermore, understanding the underlying molecular resistance mechanisms of such candidate genes will help to employ effective strategies to negate the continuous and rapid evolution of the pathogen around the globe (Li et al., 2017b). CNL genes are an important class of resistance genes that mediates plant disease resistance through direct or indirect recognition of pathogen effector proteins (Rairdan et al., 2008; Césari et al., 2014). The *Arabidopsis* CNL protein RPS2 eliminates its constitutively bound protein RIN4 to mediate the recognition of the cognate pathogen effector AvrRpt2 of *Pseudomonas syringae* leading to the activation of the RPS2-mediated signaling pathway (Michael and Staskawicz, 2003). In tomato, the CNL protein Prf interacts with Pto kinase through a unique N-terminal domain to form a high molecular weight complex, facilitating the specific recognition of *P. syringae* effector AvrPtoB (Mucyn et al., 2006; Xing et al., 2007). To date, seven rice blast effectors have been cloned. Four, including AVR-Pia, AVR-Pita, AVR1-CO39, and AVR-Pik, can be directly recognized by CNL protein or pair (Jia et al., 2000; Kanzaki et al., 2012; Cesari et al., 2013). Currently, the discovery and functional analysis of the CNL gene in cotton resistance to VW is at the preliminary stage. Besides, only one CNL resistance gene *GbRVd* has been reported in cotton to play a role in VW (Yang et al., 2016). Therefore, it is necessary to expand our understanding and further the discovery of more such CNL genes in cotton for effective utilization in conferring resistance to Verticillium wilt.

Clustering characterizes NBS-LRR gene distribution in several species, including *Arabidopsis*, sorghum, apple, blueberry, flooded gum, and cotton, mainly due to gene replication and amplification events during the evolution process to counter the co-evolution of the pathogen (Meyers et al., 2003; Arya et al., 2014; Chen et al., 2015; Christie et al., 2016; Yang and Wang, 2016; Xiang et al., 2017; Die et al., 2018). A gene cluster is a mini-library of mutations associated with gene resistance to various pathogens (Meyers et al., 2003; Ameline-Torregrosa et al., 2008; Guo et al., 2011). In this research, 141 *GbCNL* genes (ten gene clusters and two supergene clusters) were identified from 3 to 79 *G. barbadense* (Figure 1). Based on our study and similar work in other crop-pathogen systems, we predict that these *GbCNL* genes in cotton may provide resistance to various pathogens, including *V. dahliae*.

Transcriptome analysis has also been used to identify candidate genes involved in disease resistance of several species, including *G. barbadense* (Yang and Wang, 2016; Bao et al., 2019). Our transcriptome analysis showed that ten *GbCNL* genes, including six *GbCNL*s from two largest clusters (GC11 and GC12), are significantly upregulated after *V. dahliae* inoculation, supported by RT-qPCR analysis of multiple candidate genes. The expression level of seven *GbCNL*s from GC11 and GC12 was significantly upregulated in VW-resistant cotton variety but not substantially downregulated in VW susceptible cotton variety after *V. dahliae* inoculation (Figure 2). Thus, it seems likely that the *GbCNL*s from the gene clusters GC11 and GC12 are associated with the *G. barbadense* response to *V. dahliae*. In this study, VIGS was used to screen candidate genes for VW resistance. The *GbCNL130*-silenced plants inoculated with *V. dahliae* had a significant increase in fungal biomass (Figure 3), indicating that the *GbCNL130* is the candidate gene involved in VW resistance. Gain-of-function studies in *A. thaliana* ectopically overexpressing the cotton *GbCNL130* provide evidence that resistance to VW mediated by *GbCNL130* can be employed across multiple species (Figure 4). Taken together, our study reveals that the *GbCNL130* is a new CNL resistance gene with role in VW resistance in *G. barbadense*.

Previous studies have shown that plant NBS-LRR resistance proteins mediate defense responses through the specific hormone pathway (Qi and Innes, 2013). SA is a key signaling molecule against biotrophic pathogens in hormone-mediated immune responses (Glazebrook, 2005); for instance, the automatic activation of the *Arabidopsis activated disease resistance 1-like 2* (*ADR1-L2*) mutant increases the defense hormone, SA (Roberts et al., 2013). Besides, VIGS-based silencing of *GbRVd* expression in *G. barbadense* significantly reduces SA production, thus increasing susceptibility to *V. dahliae* (Yang and Wang, 2016). The classical biochemical approaches and mutation-based genetic analysis have revealed two pathways for the enzymatic SA synthesis in plants. One is the phenylalanine pathway mediated by phenylalanine ammonia lyase, and the other is the allelic acid pathway mediated by isochorismate synthase (Chen et al., 2009). Several genes, including *GbRVd*, *GhPAO*, *GbaNA1*, *Gbve1*, *GaRPL18, and G. hirsutum spm synthase* (*GhSPMS*), mediate VW resistance through the SA signaling pathway in cotton (Zhang et al., 2012; Mo et al., 2015, 2016; Yang et al., 2016; Gong et al., 2017; Li et al., 2017b). Our results provide proof that the SA signaling pathway is activated upon ectopic expression of *GbCNL130* (Figure 5). Therefore, we conclude that the *GbCNL130* gene mediates *V. dahliae* resistance in *A. thaliana via* the activation of the SA-based branch of host defense response.

PR genes are upregulated in many species after inoculation with fungi, bacteria, or viruses (van Loon et al., 2006). On the other hand, pathogen-induced accumulation of ROS not only is responsible for the direct killing of the invading pathogens, but also activates cell wall cross-linking to strengthen the plant cell wall, thus limiting pathogen infection (Vlot et al., 2009). Plant resistance proteins, including NBS-LRR proteins, recognize specific pathogenic effectors and initiate various defense responses, such as ROS accumulation and PR gene overexpression (De Young and Innes, 2006; Caplan et al., 2008). For instance, the CNL gene *Triticum aestivum Rhizoctonia cerealis resistance 1* (*TaRCR1*) mediates *Rhizoctonia cerealis* resistance by regulating ROS production and upregulating PR gene expression in wheat (Zhu et al., 2016). Overexpression of *Arachis hypogaea resistance Ralstonia solanacearum 5* (*AhRRS5*), a new peanut *NBS-LRR* gene, enhances *Ralstonia solanacearum* resistance in tobacco. *AhRRS5* overexpression also involves ROS accumulation as well as PR gene upregulation through several signaling pathways (Zhang et al., 2017). Multiple defense responses, including hormone induction, ROS accumulation, and PR gene overexpression, play an important role in cotton VW resistance (Gao et al., 2013; Zhang et al., 2013; Mo et al., 2015; Duan et al., 2016; Yang et al., 2016; Gong et al., 2017; Dhar et al., 2020). Finally, in the current study, we have employed the above predicted cotton *GbCNL130* gene in *A. thaliana* resulting in significant increase in ROS accumulation accompanied by elevated PR gene expression in response to *V. dahliae* infection. The above approach led to reduced severity of VW symptoms in *Arabidopsis*, thus establishing a cross-species conservation of its functionality and effectiveness against a challenging recalcitrant pathogen with a very wide host range (Figure 6).

## Conclusion

In this study, the CNL genes in *G. barbadense* (*GbCNL*s) were identified, and the *GbCNL*s in the two largest cluster (GC11 and GC12) were found to significantly respond to *V. dahliae* infection. VIGS analysis showed that *GbCNL130* gene silencing reduces *V. dahliae* resistance in *G. barbadense* plant, while *GbCNL130* gene overexpression significantly increased resistance in *A. thaliana*. This research also shows that the *GbCNL130* gene can improve VW resistance by activating the SA signaling pathway to mediate ROS accumulation and PR gene upregulation across different hosts. Since *V. dahliae* has a very wide host range, this discovery has potential implications for other host plant species. In summary, this study provides genetic resources for molecular genetic breeding of VW-resistant varieties not only in cotton but also in other plant hosts affected by *V. dahliae*.

## Data Availability Statement

The original contributions presented in the study are included in the article/Supplementary Material; further inquiries can be directed to the corresponding author.

## Author Contributions

TL performed the research and wrote the manuscript. QZ and XJ analyzed the data. RL and ND conceptualized and edited the manuscript. All authors approved the submitted version.

## Funding

This work was supported by the National Natural Science Foundation of China (31901866) and the Natural Science Foundation of Shandong Province (ZR2019BC090).

## Conflict of Interest

The authors declare that the research was conducted in the absence of any commercial or financial relationships that could be construed as a potential conflict of interest.

## Publisher’s Note

All claims expressed in this article are solely those of the authors and do not necessarily represent those of their affiliated organizations, or those of the publisher, the editors and the reviewers. Any product that may be evaluated in this article, or claim that may be made by its manufacturer, is not guaranteed or endorsed by the publisher.

## Footnotes

^1^ https://www.cottongen.org/

^2^ www.ebi.ac.uk/Tools/pfa/pfamscan/

^3^ http://paircoil2.csail.mit.edu/
