# Supplementary material for: Cotton CC-NBS-LRR Gene GbCNL130 Confers Resistance to Verticillium Wilt Across Different Species
Source: Front Plant Sci. 2021 Sep 8;12:695691. doi: 10.3389/fpls.2021.695691 (PMC8456104; doi:10.3389/fpls.2021.695691)
Supplement: Supplementary file 1 [file Data_Sheet_1.docx]

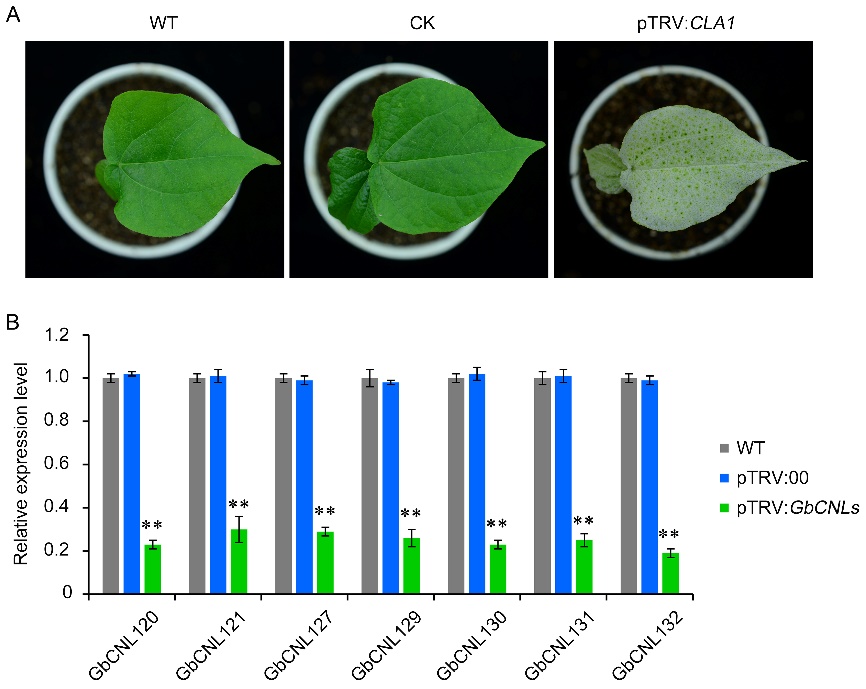


**FIGURE S1 |** Albino phenotype and detection of candidate gene silencing efficiency. (**A**) Phenotype of wild-type (WT), *pTRV2*:00 (CK) and *CLA1*-silenced of cv. Hai No. 7124 plants. (**B**) Detection of candidate genes silencing efficiency. Values represent averages of three independent biological replicates of three plants each. Double asterisk (**) indicates a significant difference at (P<0.01).


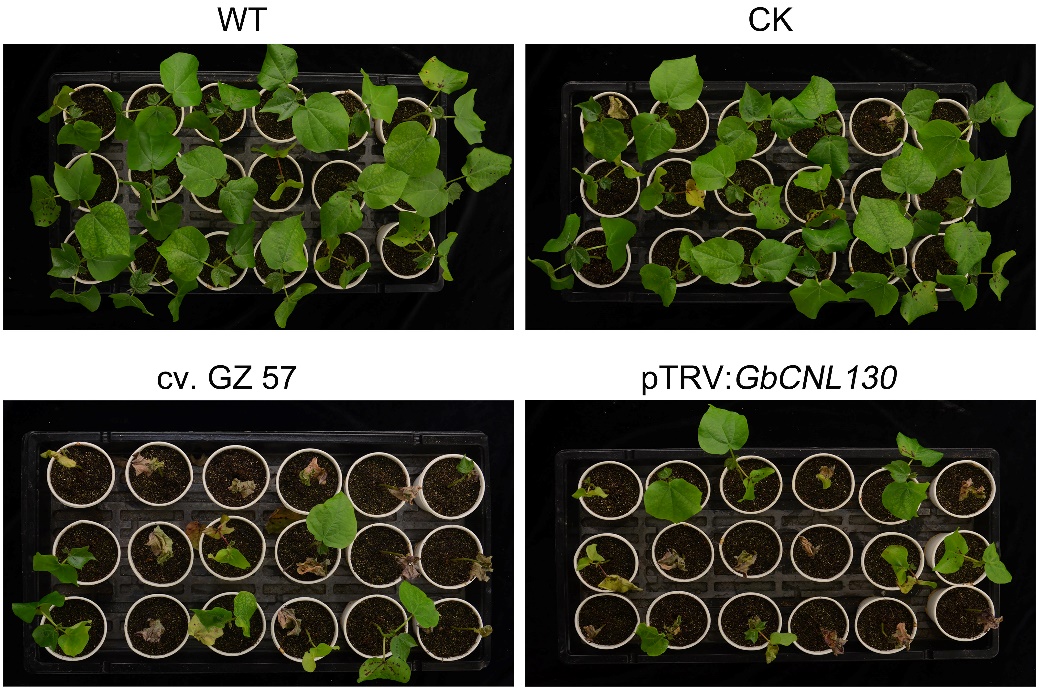


**FIGURE S2 |** The population phenotype of plants with inoculation of *Verticillium dahliae*. After 21 days of gene silencing, the wild-type (WT), *pTRV2*:00 (CK), cv. Giza No. 57 (cv. GZ 57) and *GbCNL130* gene-silenced plants were evaluated for disease resistance using the root-dip method. The resistance phenotypes were identified after three weeks.
